# Quantitative Proteomic Analysis of the Effect of Fluoride on the Acquired Enamel Pellicle

**DOI:** 10.1371/journal.pone.0042204

**Published:** 2012-08-01

**Authors:** Walter L. Siqueira, Meltem Bakkal, Yizhi Xiao, Jennifer N. Sutton, Fausto M. Mendes

**Affiliations:** 1 Department of Biochemistry and School of Dentistry, Schulich School of Medicine and Dentistry, Western University, London, Onatrio, Canada; 2 Department of Pediatric Dentistry, School of Dentistry, Marmara University, Istanbul, Turkey; 3 Thermo Fisher Scientific, Cambridge, Massachusetts, United States of America; 4 Department of Pediatric Dentistry, Faculty of Dentistry, University of São Paulo, São Paulo, São Paulo, Brazil; University of Technology Sydney, Australia

## Abstract

The acquired enamel pellicle (AEP) is a thin film formed by the selective adsorption of salivary proteins onto the enamel surface of teeth. The AEP forms a critical interface between the mineral phase of teeth (hydroxyapatite) and the oral microbial biofilm. This biofilm is the key feature responsible for the development of dental caries. Fluoride on enamel surface is well known to reduce caries by reducing the solubility of enamel to acid. Information on the effects of fluoride on AEP formation is limited. This study aimed to investigate the effects of fluoride treatment on hydroxyapatite on the subsequent formation of AEP. In addition, this study pioneered the use of label-free quantitative proteomics to better understand the composition of AEP proteins. Hydroxyapatite discs were randomly divided in 4 groups (*n* = 10 per group). Each disc was exposed to distilled water (control) or sodium fluoride solution (1, 2 or 5%) for 2 hours. Discs were then washed and immersed in human saliva for an additional 2 hours. AEP from each disc was collected and subjected to liquid chromatography electrospray ionization mass spectrometry for protein identification, characterization and quantification. A total of 45 proteins were present in all four groups, 12 proteins were exclusively present in the control group and another 19 proteins were only present in the discs treated with 5% sodium fluoride. Relative proteomic quantification was carried out for the 45 proteins observed in all four groups. Notably, the concentration of important salivary proteins, such as statherin and histatin 1, decrease with increasing levels of fluoride. It suggests that these proteins are repulsed when hydroxyapatite surface is coated with fluoride. Our data demonstrated that treatment of hydroxyapatite with fluoride (at high concentration) qualitatively and quantitatively modulates AEP formation, effects which in turn will likely impact the formation of oral biofilms.

## Introduction

Biomineralization is the process that living organisms produce and maintain mineral tissues such as bones, cartilages and teeth. The mechanism related to the formation and homeostasis of those minerals is not well understood, but there is a strong belief that proteins play an important role [1]. In addition, phenomena related to mineral crystal growth inhibition, demineralization and remineralization are always related to protein adsorption on the surface of the mineral. These events are also common in dental enamel, a crystalline calcium phosphate mineral close in composition to hydroxyapatite.

Dental caries (tooth decay) is a complex disease, characterized by demineralization of tooth structure by bacterial organic acids from the dental biofilm supported by salivary proteins adsorbed on mineral tooth surface [2]. According to the World Health Organization (WHO), dental caries remains a major public health problem in most industrialized countries, affecting 60–90% of school children and the vast majority of adults. It is also the most prevalent oral disease in several Asian and Latin American countries [3].

Fluoride is well known to reduce dental caries by reducing the solubility and enhancing remineralization of dental enamel by the incorporation of available fluoride into the tooth structure during acid attacks [4]. Basically, there are two forms to delivery and promote the benefits of fluoride on dental enamel. The self-administered methods of fluoride utilization include systemic application, for instance community water fluoridation, and topical application through daily use of fluoride dentifrices with concentration ranging from 1,000 to 1,500 ppm of fluoride. On the other hand, professional methods of fluoride utilization employ products in much higher concentration (ranging from 1% to 5% sodium fluoride). Professional fluoride applications have been effective complimentary methods to prevent and arrest caries progress, mainly in the patients with caries activity [5].

In addition to the positive effects of fluoride, several salivary proteins appear to be involved in inhibition of enamel demineralization and/or remineralization processes [6],[7],[8],[9] with a protective role that depends on the selective adsorption of these salivary proteins onto the enamel surface of teeth. These proteins attached to the enamel surface form a thin protein layer called Acquired Enamel Pellicle (AEP) [10], [11]. The AEP protects the tooth from enamel demineralization by acting as a natural diffusion barrier inhibiting direct contact between the tooth surfaces and oral bacteria acids [11], [12]. More specifically, the AEP decreases diffusion rates of phosphate and calcium ions into the surrounding fluid following exposure of the tooth to acidic conditions, thus protecting against demineralization [13]. In addition, by virtue of its location, AEP is the interface between the mineral phase of dental enamel and the plaque, a biofilm largely composed of oral bacteria [11]. This biofilm is the key feature responsible for the development of dental caries and other oral diseases such as periodontitis. Since attachment of bacteria to the AEP is the earliest event in the bacterial colonization of enamel, the protein composition of AEP plays a critical role in the modulation of oral biofilm. This oral biofilm modulation can determine the balance between oral health and disease because specific salivary proteins promote adhesion and plaque formation of pathogenic bacteria [14].

Despite the importance of both fluoride and AEP to prevent dental caries, scarce information is available on the effects of fluoride on AEP formation. It is established that minimum changes in the chemical properties of solid surfaces such as hydroxyapatite have influence on the adsorption behavior of salivary proteins to the surfaces [15], [16]. Several studies indicate that the topical use of fluoride on enamel results in the formation of a layer of calcium-fluoride (CaF_2_) like material, which strongly change the composition and chemical properties of the enamel surface [17]. The aim of this study was to investigate the effects of topical fluoride treatment on hydroxyapatite on the subsequent formation of AEP. In addition, this study pioneered the use of label-free quantitative proteomics for better understanding on the composition and function of AEP proteins.

## Methods

### Whole Saliva Collection

This study was approved by the Research Human Ethics Board of Western University (review number 16181E). Written informed consent was acquired from all subjects in this study. Whole saliva from 3 healthy subjects were collected between 9:00 and 11:00 A.M. under masticatory stimulation using Parafilm, 25 cm^2^ ∼1 g [18]. The samples were kept on ice during the collection procedure, and immediately after collection centrifuged at 14,000×*g* for 20 min at 4°C. Whole saliva supernatants were separated from the pellet, a saliva pool was carried out and used immediately for the proposed analyses. The total protein concentration of whole saliva supernatant was measured by the bicinchoninic acid (BCA) assay (Pierce Chemical, Rockford, IL, USA) with bovine serum albumin used as the standard.

### Incubation of Fluoride Treated HA Discs with Human Saliva

To evaluate the effect of sodium fluoride (NaF) on adsorption of salivary protein on hydroxyapatite (HA) surface, HA discs (5 mm diameter×1 mm thickness, Clarkson Chromatography products, Inc.) were cleaned by 5 min sonication in distilled water. Forty HA discs were randomly divided in 4 groups (n = 10 per group). NaF solutions were prepared with distilled water and NaF (ACS chemical grade, 99% pure, BDH, West Chester, PA, USA) under constant agitation at 37°C for a period of 2 hours. Each disc was exposed to 300 µl of distilled water (control group) or 1% NaF (1% NaF group), 2% NaF (2% NaF group), and 5% NaF (5% NaF group) for a period of 2 h at 37°C with gentle agitation. After this period, the discs were washed in distilled water for 30 sec. Subsequently, the pre-treated discs were immersed into individual vials containing 100 µg of whole saliva supernatant for an additional period of 2 h at 37°C. Immediately after protein incubation period the discs were extensively washed using distilled water to remove any weak binding salivary protein.

### Harvesting of *in vitro* AEP

AEP proteins formed on each HA disc over a 2 h period was collected by incubating with 150 µl of a solution containing 80% acetonitrile, 19.9% water and 0.1% TFA and sonicated for 5 min. This procedure was repeated three times to release all the mineral-associated protein or peptides for subsequent analyses and the 450 µl combined in a vial/disc. Eluted AEP material from each of the ten discs from the same group was pooled and concentrated by a rotary evaporator. The total protein concentration was assessed by the Micro Bicinchoninic acid (Micro BCA) assay.

### In–solution Digestion

Equal protein amount (10 µg) from both experimental and control groups were dried by a rotary evaporator, denatured and reduced for 2 h by the addition of 200 µl of 4 M urea, 10 mM dithiothreitol (DTT), and 50 mM NH_4_HCO_3_, pH 7.8. After four-fold dilution with 50 mM NH_4_HCO_3_, pH 7.8, tryptic digestion was carried out for 18 h at 37°C, after the addition of 2% (w/w) sequencing-grade trypsin (Promega, Madison, WI, USA).

### Liquid Chromatography Electrospray Ionization Tandem Mass Spectrometry (LC-ESI-MS/MS)

Peptide separation and mass spectrometric analyses were carried out with a nano-HPLC Proxeon (Thermo Scientific, San Jose, CA, USA) which allows in-line liquid chromatography with the capillary column, 75 µm×10 cm (Pico Tip™ EMITTER, New Objective, Woburn, MA) packed in-house using Magic C18 resin of 5 µm diameter and 200 Å pores size (Michrom BioResources, Auburn, CA) linked to mass spectrometer (LTQ-Velos, Thermo Scientific, San Jose, CA, USA) using an electrospray ionization in a survey scan in the range of m/z values 390–2000 tandem MS/MS. Equal amount of all samples were dried by rotary evaporator and re-suspended in 20 µl of 97.5 % H_2_O/2.4% acetonitrile/0.1% formic acid and then subjected to reversed-phase LC-ESI-MS/MS. The nano-flow reversed-phase HPLC was developed with linear 80 minutes gradient ranging from 5% to 55% of solvent B in 65 minutes (97.5% acetonitrile, 0.1% formic acid) at a flow rate of 300 nl/min with a maximum pressure of 280 bar. Electrospray voltage and the temperature of the ion transfer capillary were 1.8 kV and 250°C respectively. Each survey scan (MS) was followed by automated sequential selection of seven peptides for CID, with dynamic exclusion of the previously selected ions.

The obtained MS/MS spectra were searched against human protein databases (Swiss Prot and TrEMBL, Swiss Institute of Bioinformatics, Geneva, Switzerland, http://ca.expasy.org/sprot/) using SEQUEST algorithm in Proteome Discoverer 1.3 software (Thermo Scientific, San Jose, CA, USA). Search results were filtered for a False Discovery rate of 1% employing a decoy search strategy utilizing a reverse database. An addition inclusion criterion for positive identification of proteins was that the same protein passing the filter score from at least in three different MS analyses from the same group in a total of four MS analyses per group.

### Integration and Relative Proteome Quantitation

For quantitative proteome analysis, three MS raw files from each pooled group were analyzed using SIEVE software (Version 2.0 Thermo Scientific, San Jose, CA, USA). Signal processing was performed in a total of 12 MS raw files. The SIEVE experimental workflow was defined as “Control Compare Trend Analysis” where one class of samples are compared to one or more other class of samples. Here the control samples were compared to each of the samples that were treated with different percentages of NaF (1%, 2% and 5%). For the alignment step, a single MS raw file belonging to the HA group was selected as the reference file and all of the other files were adjusted to generate the best correlation to this reference file. After alignment, the feature detection and integration (or framing) process was performed using the MS level data with a feature called “Frames From MS2 Scans” only. When using this type of framing only MS mass-to-charge ratio (m/z) values that were associated with MS2 scan are used. Any m/z measurements that do not have MS2 were ignored. The parameters used consisted of a frame m/z width of 1500 ppm and a retention time width of 1.75 min. A total of 216,099 MS2 scans were present in all of the 12 RAW files that resulted in a total of 20,158 frames. Then peak integration was performed for each frame and these values were used for statistic analysis. Next, peptide sequences obtained from the database search using SEQUEST algorithm in Proteome Discoverer 1.3 were imported into SIEVE. A filter was applied to the peptide sequences during the import that eliminated all sequences with a Percolator q-value greater than 1% (false discovery rate). Peptides were grouped into proteins and a protein ratio and p-value were calculated. SIEVE uses a weighted average of the peptide intensities for the protein calculation. By using the weighted average, peptides with lower variance in their intensity measurements have a higher weight on the overall protein ratio. This is done to decrease variance in protein level quantities based on variance of the peptides that compose proteins. Only proteins observed in all four groups were quantified. HA group was used as our default group and all other three groups were compared with HA control group.

Relative abundance of an individual protein from HA group was considered significantly different protein level when the values observed were <0.75 for decrease abundance or > 1.25 for increase abundance, and a *p*-value <0.05 as described [19].

### X-ray Photoelectron Spectroscopy (XPS)

The XPS analyses were carried out with a Kratos Axis Ultra spectrometer using a monochromatic Al K(alpha) source (15 mA, 14 kV). XPS can detect all elements except hydrogen and helium, probes the surface of the sample to a depth of 5–10 nm, and has detection limits ranging from 0.1 to 0.5 atomic percent depending on the element. The instrument work function was calibrated to give a binding energy (BE) of 83.96 eV for the Au 4f7/2 line for metallic gold and the spectrometer dispersion was adjusted to give a BE of 932.62 eV for the Cu 2p3/2 line of metallic copper. The Kratos charge neutralizer system was used on all specimens. Survey scan analyses were carried out with an analysis area of 300×700 µm and a pass energy of 160 eV. Spectra were analyzed using CasaXPS software (version 2.3.14).

### Enzyme-linked Immunosorbent Assay (ELISA)

ELISA microtiter plate (96-wells) was coated with 100 µl of AEP protein material from each group (10 µg/ml) at 37°C for 1 hour. The plate was then washed three times with 250 µl Tris Buffered Saline (TBS) per well and 200 µl TBST containing 3% BSA added to each well to block uncoated sites, and incubated overnight at 4°C. Primary anti statherin antibody (50 µl; 1∶1000 dilution, Abcam, ab97950, MA, USA) in TBST containing 1% BSA was added to each well and incubated at 37°C for 1.5 h, followed by washing three times, and incubation with horse radish peroxidase (HRP) linked anti-rabbit IgG antibody (100 µl; 1∶30000 dilution, ROCKLAND, PA, USA) in TBST containing 1% BSA. After incubation in the dark for 1 h at room temperature OPD (o-phenylenediamine dihydrochloride, Sigma-Aldrich, MO, USA) was added and product wasanalyzed spectrophotometrically at 490 nm. Statherin levels in each sample were determined by reference to a statherin standard curve and assessed by linear regression analysis. Statherin protein (Chinapeptide, Shanghai, China) purity and M^r^ 5380 were determined by mass spectrometry analysis. Analysis of variance and Student-Newman-Keuls test for pairwise comparisons was carried out to compare the values among the groups.

## Results

### HA Surface Characterization

**Figure 1:**
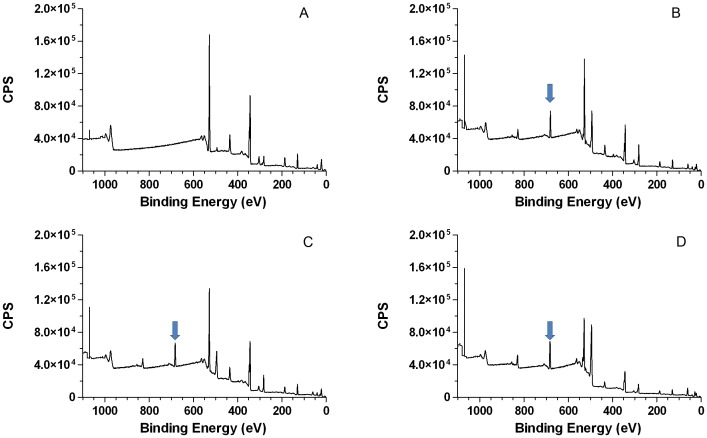
XPS wide scan spectrum of HA surface disc (**A**) **and HA surface disc pre-treated with 1 % NaF** (**B**)**, 2% NaF** (**C**) **and 5% NaF** (**D**)**.** Note: arrow indicates the fluoride peak at 685 eV binding energy.

XPS analyses of HA group and 5% NaF group are shown in Figure 1. The widescan spectrum of the control specimen shows the presence of calcium (342 eV) and phosphorus (128 eV). Treatment of the HA disc with 5% NaF shows additional fluoride peak at 685 eV binding energy. This binding energy at 685 eV supports the contention that fluoride is bound to calcium as CaF_2_ on the enamel surface. When the fluoride atoms % on the surface was measured and compared among the three groups that were treated with fluoride, the values were (mean ± standard deviation) 9.1±1.0 9.80.7 and 14.30.8 for groups 1% NaF, 2% NaF and 5% NaF respectively (Table 1). Atoms % of calcium and phosphorus were also acquired and Ca/P ratio were calculated to each group (Table 1).

**Table 1:** Atoms % of Calcium, Fluoride and Phosphorus on the HA surface treated with NaF.

	Calcium		Fluoride	Phosphorus	Ratio Calcium/Phosphorus
HA		16.8±0.4		9.8±0.7	1.71
1% NaF		10.2±1.4	9.1±1.0	6.2±0.6	1.64
2% NaF		12.2±0.3	9.8±0.7	7.9±0.7	1.55
5% NaF		6.7±0.2	14.3±0.8	5.2±0.4	1.29

Mean ±SD.

### AEP Proteome Identification and Quantification

**Figure 2:**
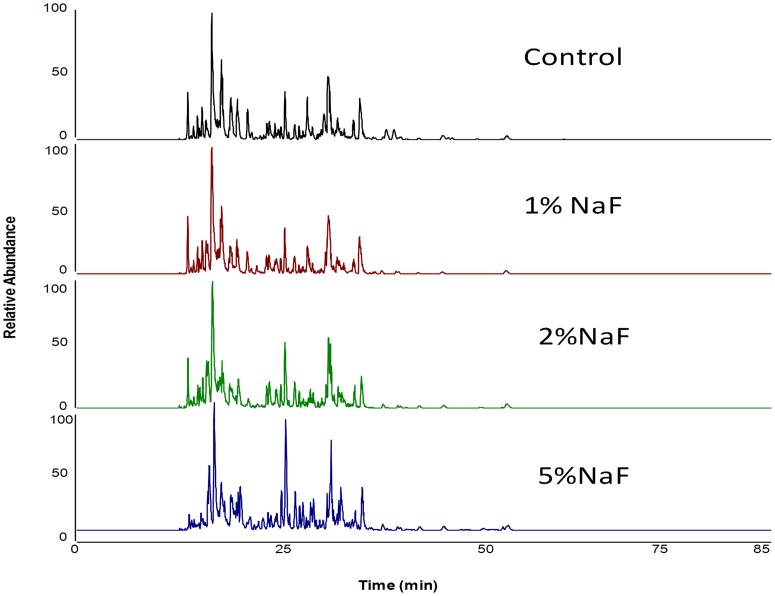
Examples of base-peak chromatograms of each studied group. Peptide separation was achieved using a nano-flow reverse-phase HPLC column, with gradient elution ranging from 5 to 55% solvent B in 65 min.

**Figure 3:**
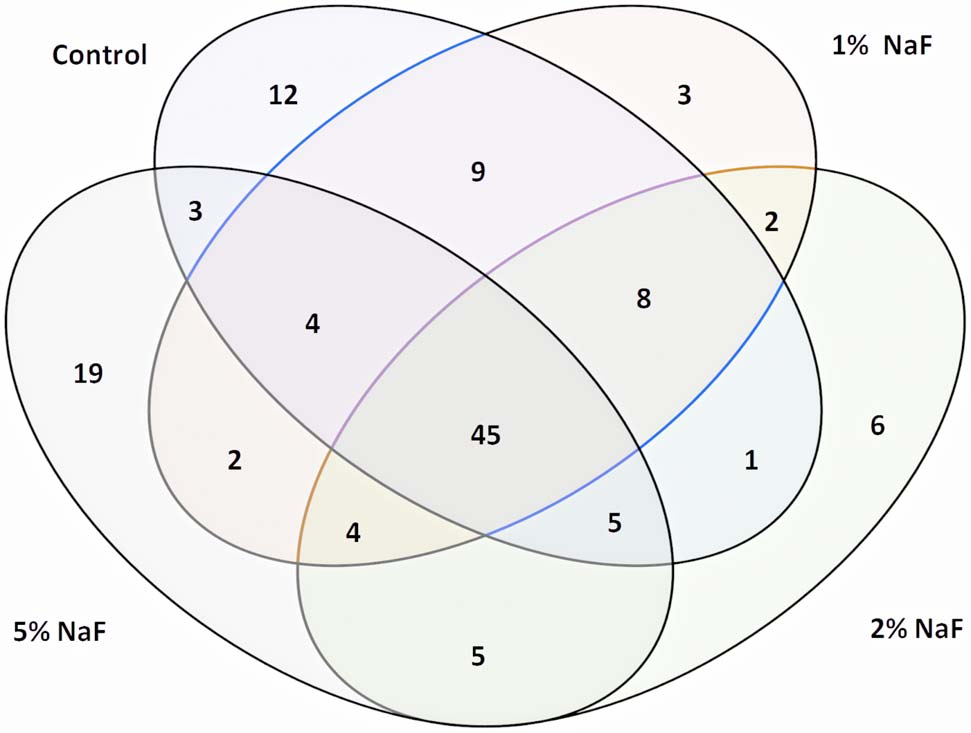
Venn diagram of AEP proteins identified in each group and across groups.

After AEP elution from HA surface and trypsinization, equal amount of peptides were subjected to nanoscale LC-ESI-MS/MS. A total of 4 runs per group were carried out. The base-peak chromatogram for reversed-phase chromatography monitored by the mass spectrometer represents the intensity of all peptide ions in the sample in a single scan. AEP proteome from all four different groups showed a consistent elution of protein/peptides range from 12 to 45 min (Figure 2). The peptide ions were identified by the SEQUEST search following the criteria as described in Methods. For the proteome identification of the AEP formed on all four different conditions carried out in this study a total of 87 different proteins were identified in HA control group, 77 different proteins were identified in 1% NaF group, 76 different proteins were identified in 2% NaF group and 87 different proteins were identified in 5% NaF group (Table 2). The majority of the proteins were identified in all four groups indicating a high overlap in AEP proteins (Table 2). Figure 3 shows a Venn diagram with the number of proteins from each group and their overlaps among the four groups. A total of 45 proteins were present in all four groups. 12 proteins were exclusively present in HA control group. 3 proteins were exclusively present in 1% NaF group. 6 proteins were exclusively present in 2% NaF group and other 19 proteins only present in 5% NaF (Table 2; Figure 3).

**Table 2:** Salivary proteins identified on hydroxyapatite discs and hydroxyapatite discs treated with 1%, 2% and 5% NaF.

	Acession number	Protein name
**Proteins present in all groups**	B1AN48	Small proline-rich protein 3
	P01036	Cystatin-S
	P01037	Cystatin-SN
	P01620	Ig kappa chain V-III region SIE
	P01777	Ig heavy chain V-III region TEI
	P01781	Ig heavy chain V-III region GAL
	P01833	Polymeric immunoglobulin receptor
	P01834	Ig kappa chain C region
	P01857	Ig gamma-1 chain C region
	P01871	Ig mu chain C region
	P01876	Ig alpha-1 chain C region
	P01877	Ig alpha-2 chain C region
	P02808	Statherin
	P02812	Basic salivary proline-rich protein 2
	P02814	Submaxillary gland androgen-regulated protein 3B
	P04075	Fructose-bisphosphate aldolase A
	P04080	Cystatin-B
	P04264	Keratin, type II
	P04745	Alpha-amylase 1
	P05109	Protein S100-A8
	P06702	Protein S100-A9
	P06733	Alpha-enolase
	P09211	Glutathione S-transferase
	P09228	Cystatin-SA
	P0CG05	Ig lambda-2 chain C regions
	P11021	78 kDa glucose-regulated protein
	P12273	Prolactin-inducible protein
	P13645	Keratin, type I
	P15515	Histatin-1
	P23284	Peptidyl-prolyl cis-trans isomerase B
	P25311	Zinc-alpha-2-glycoprotein
	P29508	Serpin B3
	P30740	Leukocyte elastase inhibitor
	P31025	Lipocalin-1
	P35527	Keratin, type I cytoskeletal 9
	P54108	Cysteine-rich secretory protein 3
	P61626	Lysozyme C
	Q96DA0	Zymogen granule protein 16
	B7Z4X2	Lactotransferrin
	E7EMJ3	Lactoperoxidase
	E7EMQ1	Carbonic anhydrase VI
	E7EQ46	Uncharacterized protein
	P02768	Albumin
	E9PBV3	Suprabasin
	F5GXH2	Lactate dehydrogenase A
**Proteins present in HA, 1%** **and 2% NaF groups**	B1AHN5	Hsp70 interacting protein
	P01009	Serpin A1
	P01605	Ig kappa chain V-I region
	P02810	Salivary acidic proline-rich phosphoprotein 1
	P27482	Calmodulin 3
	Q8IUK7	ALB protein
	F5GYT4	Actin, beta
	F5H0L3	6-phosphogluconate dehydrogenase
**Proteins present in HA, 2% and 5% NaF groups**	B4DRT4	Phosphatidylethanolamine-binding protein 1
	P02533	Cytokeratin-14
	P0CG04	Ig lambda-1 chain C regions
	P13647	Keratin, type II cytoskeletal 5
	P35908	Keratin, type II cytoskeletal 2
**Proteins present in HA, 1% and 5% NaF groups**	B1ALW1	Thioredoxin
	Q08188	Protein-glutamine gamma-glutamyltransferase
	Q8TDL5	Long palate, lung and nasal epithelium carcinoma-associated protein 1
	E9PKG6	Nucleobindin 2
**Proteins present in HA and 1% NaF groups**	E7EUT5	Glyceraldehyde-3-phosphate dehydrogenase
	P01034	Cystatin-C
	P02787	Serotransferrin
	P08493	Matrix Gla protein
	P27797	Calreticulin
	P81605	Preproteolysin
	Q13765	NAC-alpha
	Q6P5S2	protein C6orf58
	P37802	transgelin-2
**Protein present in HA and 2% NaF groups**	P08779	Keratin, type I cytoskeletal 16
**Proteins present in HA and 5% NaF groups**	P01625	Ig kappa chain V-IV region Len
	Q86YZ3	Hornerin
	Q8IWZ5	Tripartite motif-containing protein 42
**Proteins exclusively present in control group**	O00391	Sulfhydryl oxidase 1
	P01597	Ig kappa chain V-I region
	P15924	Desmoplakin
	P31949	Protein S100-A11
	P62158	Calmodulin
	Q02413	Desmoglein-1
	Q16739	Ceramide glucosyltransferase
	Q6ZVX7	Non-specific cytotoxic cell receptor protein 1 homolog
	Q8TDN4	Ik3-1
	C9J0E4	Cystatin A (stefin A)
	E5RGE1	zeta polypeptide
	E9PHI2	Heat shock protein 90 kDa alpha
**Proteins present in 1%, 2% and 5% NaF groups**	P01040	Cystatin-A
	Q9HC84	Mucin-5B
	F5GZ39	Ubiquitin C
	F5H805	Uncharacterized protein
**Proteins present in 1% and 2% NaF groups**	P07108	Acyl-CoA-binding protein
	Q01469	Fatty acid-binding protein
**Proteins present in 1% and 5% NaF groups**	Q96DR5	Short palate, lung and nasal epithelium carcinoma-associated protein 2
	E7EPA8	Prolyl 4-hydroxylase, beta polypeptide
**Proteins exclusively present in 1% NaF group**	P20930	Filaggrin
	D6R9A6	High mobility group protein B2
	E9PLJ3	Cofilin 1
**Proteins present in 2% and 5% NaF groups**	P01593	Ig kappa chain V-I region AG
	P01617	Ig kappa chain V-II region TEW
	P01765	Ig heavy chain V-III region TIL
	P18510	Interleukin-1 receptor antagonist protein
	E7EQR4	Ezrin
**Proteins exclusively present in 2% NaF group**	B4E1H9	Phosphoglycerate kinase
	P16401	histone H1.5
	P80748	Ig lambda chain V-III region
	Q567Q0	Cyclophilin A
	E7EPW1	Uncharacterized protein
	B4DUA5	Uncharacterized protein
**Proteins exclusively present in 5% NaF group**	E9PF79	Pyruvate kinase
	P00558	Phosphoglycerate kinase 1
	P04083	Annexin A1
	P04406	Glyceraldehyde-3-phosphate dehydrogenase
	P05164	Myeloperoxidase
	P07737	Profilin-1
	P17931	Galectin-3
	P23528	Cofilin-1
	P26038	Moesin
	P30040	Endoplasmic reticulum resident protein 29
	P52209	6-phosphogluconate dehydrogenase, decarboxylating
	Q5SX91	GDP dissociation inhibitor 2 (Fragment)
	Q5T8F0	Cathepsin L1
	C9J8F3	Uncharacterized protein
	C9JMC5	Uncharacterized protein
	E7EQL7	Uncharacterized protein
	E9PFF2	Transketolase
	F5H0N0	Actin, gamma 1
	F5H8B6	Proteinase 3

Relative proteomic quantification was carried out in the 45 proteins observed in all four groups. Differential display of MS/MS spectra was carried out using SIEVE software. A threshold for significant differential level was set up at variation higher or lower than the 25% protein level observed in the control group. None of the 45 proteins show a differential level between HA control group and 1% NaF group. On the other hand, 14 proteins showed a decrease level between HA control group and 2% NaF group and 1 protein showed an increase level. Comparison of the HA control group with 5% NaF group, demonstrated 19 proteins with a reduced protein level while 1 protein showed an increase (Table 3).

**Table 3:** AEP protein abundance ratios from 1% NaF, 2% NaF and 5% NaF groups.

Accession Number	Protein Name	Ratio 1%NaF/HA	p	Ratio 2%NaF/HA	p	Ratio 5%NaF/HA	p
P01620	Ig kappa chain V-III region SIE	1.11	0.605	1.09	0.001	0.83	0.154
B1AN48	Small proline-rich protein 3	1.06	0.326	0.81	0.001	0.77	0.001
B7Z4X2	highly similar to Lactotransferrin	0.79	0.001	0.84	0.001	0.88	0.001
E7EMJ3	Lactoperoxidase	0.88	0.514	0.38	0.001	0.02	0.001
E7EMQ1	Carbonic anhydrase 6	0.77	0.240	0.76	0.064	0.63	0.014
E7EQ46	Uncharacterized protein	0.93	0.989	0.89	0.006	1.02	0.374
P02768	Serum albumin	0.85	0.132	0.67	0.001	0.62	0.001
E9PBV3	Suprabasin	1.15	0.239	1.22	0.191	0.94	0.093
F5GXH2	Lactate dehydrogenase A	0.85	0.269	0.94	0.004	0.86	0.001
P01036	Cystatin S	0.90	0.001	0.65	0.001	0.73	0.001
P01037	Cystatin SN	0.77	0.001	0.62	0.001	0.56	0.001
P01777	Ig heavy chain VIII region TEI	0.89	0.847	0.82	0.006	0.71	0.001
P01781	Ig heavy chain VIII region GAL	1.01	0.968	0.94	0.896	1.09	0.966
P01833	Polymeric immunoglobulin receptor	0.96	0.366	0.95	0.245	1.04	0.118
P01834	Ig kappa chain C region	0.85	0.001	0.88	0.001	0.78	0.001
P01857	Ig gamma 1 chain C region	0.94	0.256	0.81	0.068	0.78	0.001
P01871	Ig mu chain C region	1.11	0.496	0.96	0.992	1.14	0.719
P01876	Ig alpha 1 chain C region	1.01	1.000	0.96	0.037	1.04	0.352
P01877	Ig alpha 2 chain C region	1.00	0.279	0.84	0.058	0.83	0.001
P02808	Statherin	0.86	0.999	0.44	0.001	0.38	0.001
P02812	Basic salivary proline rich protein 2	1.12	0.008	1.35	0.001	1.88	0.004
P02814	Proline-rich protein 3	0.76	0.001	0.83	0.013	0.72	0.001
P04075	Fructose bisphosphate aldolase A	1.05	0.528	0.91	0.173	0.95	0.317
P04080	Cystatin B	0.90	0.510	0.88	0.329	0.72	0.001
P04264	Keratin type II cytoskeletal 1	0.54	0.001	0.60	0.001	0.72	0.001
P04745	Alpha amylase 1	0.95	0.935	0.60	0.049	0.72	0.004
P05109	Protein S100A8	1.07	0.163	0.94	0.001	1.25	0.001
P06702	Protein S100A9	1.16	0.001	1.03	0.001	1.04	0.001
P06733	Alpha enolase	0.75	0.001	0.62	0.001	0.49	0.001
P09211	Glutathione S transferase P	0.85	0.586	0.76	0.039	0.81	0.122
P09228	Cystatin SA	1.09	0.002	0.93	0.010	0.69	0.001
P0CG05	Ig lambda-2 chain C regions	0.92	0.001	0.83	0.935	0.96	0.222
P11021	78 kDa glucose regulated protein	1.01	0.570	0.88	0.380	0.96	0.078
P12273	Prolactin inducible protein	0.86	0.986	0.86	0.933	0.89	0.461
P13645	Keratin_ type I cytoskeletal 10	0.66	0.000	0.61	0.001	0.60	0.001
P15515	Histatin 1	0.90	0.001	0.60	0.001	0.58	0.001
P23284	Peptidylprolyl cistrans isomerase B	1.10	0.001	1.01	0.993	1.06	0.513
P25311	Zinc alpha 2 glycoprotein	0.99	1.000	0.85	0.290	0.95	1.000
P29508	Serpin B3	0.94	0.422	0.87	0.187	1.01	0.632
P30740	Leukocyte elastase inhibitor	0.96	0.905	0.79	0.001	1.02	0.141
P31025	Lipocalin 1	0.92	0.808	0.83	0.006	0.97	0.964
P35527	Keratin type I cytoskeletal 9	0.57	0.000	0.55	0.001	0.68	0.001
P54108	Cysteine rich secretory protein 3	1.00	0.013	0.69	0.001	0.65	0.001
P61626	Lysozyme C	0.72	0.000	0.59	0.001	0.48	0.001
Q96DA0	Zymogen granule protein 16 homolog B	0.79	0.250	0.70	0.002	0.52	0.002

Note. Ratio HA/HA was used as reference and the value for all proteins is 1.000. p: statistical p-value.

**Figure 4:**
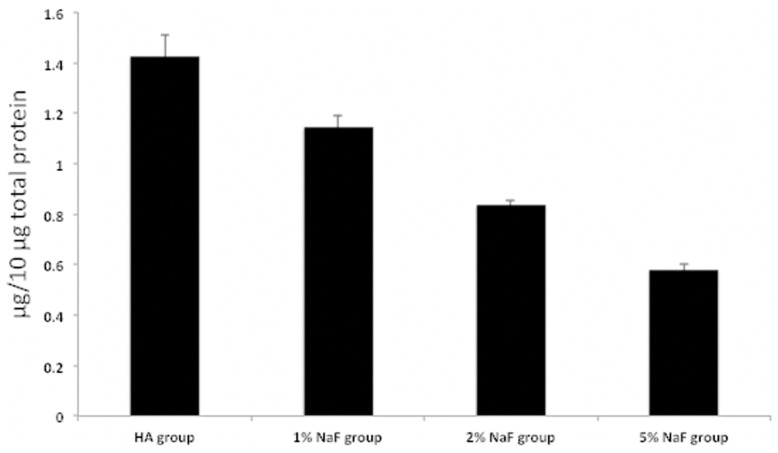
ELISA experiment with 10 µg/ml of AEP material for each group and anti-statherin antibody. Each bar represents the mean value ±SD of one experiment performed in triplicate.

To validate the differential protein level identified by quantitative mass spectrometry approach, ELISA was carried out on statherin, the protein with well-known characteristics and functions in the oral cavity. By MS a significant reduced protein level was observed at high fluoride concentration compared to control HA-discs (Table 3). By ELISA statherin contents were demonstrated to decrease from 1.43±0.08 µg/10 µg AEP total protein in the control HA disc, to 1.15±0.05, 0.78±0.06 and 0.58±0.02 in the 1%, 2%, 5% NaF groups, respectively (Figure 4).

## Discussion

Topical fluoride treatment of the enamel surface may alter the chemical properties of the enamel surface by means of a Ca-F_2_ material covering the enamel surface [20]. It was our observation that HA surface changes after the treatment with fluoride. XPS showed that HA incubated with fluoride change the atoms % on the surface (Figure 1). It was not surprise that according to the concentration of fluoride increase in the sample group, the atoms % of fluoride increase in the surface (Table 1).

It is known that HA binds proteins through both calcium (positive) and phosphate (negative) sites on the surface [21], [22]. The previous studies using classical biochemical assays examined the binding and selectivity of salivary proteins on HA. As one of the major outcomes, phosphorylated and negative salivary proteins such as statherin, histatin 1, and acidic proline-rich protein (acidic PRP) have shown a strong affinity to HA and they are considered pellicle precursor proteins [23], [24], [25]. In addition, few studies had investigated the AEP composition of HA coated with fluoride. For example, amino acid analyses showed no major differences in the amino acid compositions between AEP collected from fluoride covered enamel and AEP collected from regular enamel [26] , but the salivary protein adsorption on enamel treated with fluoride was considerably reduced compared to the adsorption on untreated enamel surface [26], [27]. Recent developments of sensitive proteomic methodologies have opened new avenues for the characterization of very-low-abundance biological samples such as AEP. Using this proteomic technology, we have used mass spectrometry to perform the first global proteome of human *in vivo* AEP. We identified 130 AEP proteins, which have been characterized according to origin, putative biological function, and possible role in AEP structure [28]. In the present study we explored the use of this mass spectrometry technology in conjunction with proteome quantification approach to investigate the effects of fluoride treatment on HA on the subsequent formation of AEP.

AEP proteome from all four different treatment groups showed a consistent elution of protein/peptides over the range from 12 to 45 minutes (Figure 2), demonstrating a similarity among AEP protein/peptides presented in all four groups studied. This observation is in agreement with our previous *in vivo* AEP investigation where the majority of the peptides eluted with a similar retention time [28], [29] but the number of proteins identified here was less than in our previous *in vivo* AEP study. This discrepancy probably is due to the different proteomic approach utilized before. In relation to protein identification, however, there were a significant percentage of proteins (14%) that were identified only in the HA control group but was not found in any of the groups treated with NaF. On the other hand, 21% of the proteins identified in 5% NaF group was also not found in any other groups (Table 2). This data reinforces the concept that AEP formation is based on selective adsorption of salivary proteins on enamel surface, since when we modified the HA surface with treatment of fluoride, the protein profile observed was also changed. Another very interesting observation was the absence of acidic PRP 1 in samples from 5 % NaF group. Acidic PRPs are charge negative salivary proteins that have high affinity to HA and are potent inhibitors of secondary calcium phosphate precipitation, which is in large part due to their two phosphate groups linked covalently to Ser residues in positions 8 and 22. Similar to acidic PRP 1, calmodulin which is a small calcium binding acidic protein with a ∼Mr of 17 kDa, was also not observed in the 5% NaF group. The importance of these observations is related to previous studies where acidic PRP 1 on HA promote bacterial adhesion of streptococcus mutans and actinomyces viscosus, both cariogenic and periodontal pathogenic bacteria respectively [30], [31]. In the same aspect, calmodulin is responsible for binding to Candida albicans, which is a major fungus related to oral candidiasis [32]. This data suggests that treatment of HA with fluoride (at high concentration) qualitatively modulates AEP contents that may have a role in lowering the cariogenic potential of the AEP, not only because of decrease solubility of the HA but its reduced potential to support pathogenic bacteria.

In general, the most common used techniques for quantitative proteomic analyses are 2D-PAGE (two-dimensional gel electrophoresis) and/or 2DIGE (two-dimensional differential gel electrophoresis). However, these methods are limited in sensitivity, low protein dynamic range, reproducibility and visualization of proteins in the gel [33]. Substitute techniques to 2D-PAGE and 2DIGE are non-PAGE procedures based on liquid chromatography, where relative or absolute quantification is performed using parameters of chromatogram and mass spectrometer. In addition, limited success for the quantification of proteins has been accomplished by using stable isotope labeling such as ICAT, SILAC, and iTRAQ. One of the main limitations of these methods is that full labeling of the proteins/peptides is rarely achieved and that different peptides incorporate the label at different rates that complicates data analysis.

**Figure 5:**
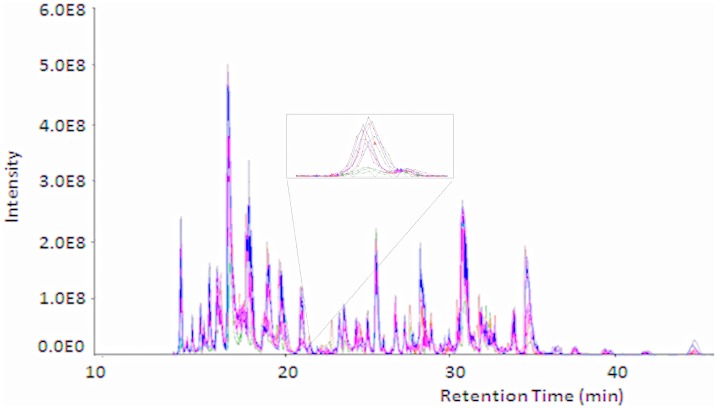
Alignment results from 12 base-peak chromatograms (3 base-peak chromatograms per group) generated by SIEVE. Inset: example of an extracted mass chromatogram for a peptide whose abundance is increased in HA (Blue), 1% NaF(red), 2% NaF (violet) groups and decreased in 5% NaF group (green).

Recently, label-free quantitation methods to be carried out in complex biological fluids such as saliva or blood are becoming more reliable [34]. Label-free quantitative proteomics provides an alternative approach for studies of AEP formation under effect of different conditions providing a more comprehensive view of composition and function of AEP proteins. In this study, we pioneered the use of a label-free LC-ESI-MS/MS methodology to explore the relative quantitation of *in vitro* AEP formed on HA disc treated with different concentrations of fluoride. After AEP proteome identified and characterized in all four different groups, SIEVE technology was used to compare the AEP protein profile. Briefly, SIEVE is a label-free-differential expression package software that aligns the MS spectra over time from different experimental conditions (HA and NaF groups) and then determines feature in the data (m/z and retention time pairs) that differ across the different conditions [35]. A first step in the quantitative proteomic analysis by SIEVE is to promote an alignment of all mass spectrometry chromatograms (Figure 5). One of the mass spectrometry chromatogram is taking as default chromatogram. All other chromatograms will be compared with the default one. In our study, we selected a default chromatogram as one of the chromatograms from HA group. Thus, all other chromatograms were compared with the selected chromatogram from HA group. Coefficient correlation score values were acquired for each mass spectrometric chromatogram and mean score values were calculated for each group. The values were 0.858 to 1% NaF group, 0.859 to 2% NaF group and, 0.805 to 5% NaF. Interestingly, we observed that according to the concentration of fluoride increase in the sample group, the alignment value will become more distant than the default chromatogram from HA group (established score 1). This observation suggests a change in quantity and quality of protein/peptides according to the concentration of fluoride.

Relative proteomic quantification was carried out for the 45 proteins observed in all four groups. Notably, all 45 proteins showed no significant variation when the concentration of fluoride was 1% on the HA surface. On the other hand, it was surprising to found that 33% of those proteins show a significant difference protein level (higher or lower) in the group treated with 2% NaF and this value was increased to 45% when the HA was treated with 5% NaF. A detailed analysis of this AEP quantitative data shows that phosphorylated and negative salivary proteins with a crucial role on tooth enamel homeostasis were drastically affected with the incorporation of fluoride on the HA surface. We saw that according to the fluoride concentration was increased on the HA surface, the protein level of these proteins were reduced. For instance, values of statherin and histatin 1 between HA group and 5% NaF group were reduced by 62% and 42%, respectively (Table 3). This shows a significant low abundance of statherin and histatin 1 when HA surface is coated with high concentration of fluoride. Our observation could be compared with the previous investigation, which shows that pre-treated HA crystals with high concentration fluoride blocked the positive charges (calcium) on the apatite surface resulting in reduced amelogenin binding, a phosphorylated and negative protein which is the dominant protein in the developing enamel matrix [36]. The importance of our observation is related to the function of these proteins. Statherin is a small molecular weight salivary protein with negative net charges that contain vicinal phosphoserine residues in positions 2 and 3. Statherin is the only salivary protein that inhibits both primary calcium phosphate precipitation (spontaneous precipitation) and secondary calcium phosphate precipitation (crystal growth), crucial functions related for the controlling of dental calculus formation and remineralization of early dental caries [37]. Beside, it has been demonstrated that statherin facilitates the binding of Actinomyces viscosus [30], [38], [39] and Fusobacterium nucleatum [40] to HA pre-coated with statherin indicating that statherin is determinant of initial microbial colonisation of tooth surface. Similar to statherin, histatin 1 is a salivary protein that exhibits the most varied of activities studied to date. The postulated *in vivo* functions for histatin 1 include buffering, modulation of mineral formation, as well as strong antifungal and antibacterial activities [41], [42]. In addition, a new feature for histatins has recently been identified. When histatins are adsorbed onto the enamel surface forming the AEP, these proteins provide protection against acid injury [6]. Recent findings have shown that, in the *in vivo* AEP, protein film that naturally cover the tooth enamel surface, significant numbers of histatins (histatins 1, 3, and 5) remain intact [6]. This observation suggests that the binding of intact histatins to the enamel surface confers the significant resistance against proteolytic degradation [43].

Only one protein shows increase level affected with the incorporation of fluoride on the HA surface. Basic Proline Rich Protein 2 (basic PRP2) was the only protein that demonstrated a significant increased protein level when HA group was compared with 2% NaF or 5% NaF groups. Interestingly, basic PRP2 has huge difference in physical-chemical characteristics than statherin and histatin 1. Basic PRP 2 is a salivary protein with significant amount of proline in its amino acid chain, non-phosphorylated and with an isoeletric point around 11.3. The function of basic PRPs is not well established in the oral cavity. However, those proteins have high affinity for dietary polyphenolic compounds (tannins) that probably provide protective effect against the potential deleterious action of these substances [44].

Understanding the composition-structure of AEP in HA surfaces treated with fluoride provide a new concept in the progress of oral diseases based on dental biofilm development such as dental caries and/or periodontal disease. Our results, despite the limitations of an *in vitro* study, presents new insights into the architecture of AEP on HA treated with fluoride and visualizes the quantitative and qualitative proteome modulation of this important tooth integument and consequently, the development of oral biofilm.
